# The Anti-Breast Cancer Stem Cell Potency of Copper(I)-Non-Steroidal Anti-Inflammatory Drug Complexes

**DOI:** 10.3390/molecules28176401

**Published:** 2023-09-01

**Authors:** Alice Johnson, Xiao Feng, Kuldip Singh, Fabrizio Ortu, Kogularamanan Suntharalingam

**Affiliations:** 1 School of Chemistry, University of Leicester, Leicester LE1 7RH, UK; alice.johnson@shu.ac.uk (A.J.); xf40@leicester.ac.uk (X.F.); ks42@leicester.ac.uk (K.S.); 2 Biomolecular Sciences Research Centre, Sheffield Hallam University, Sheffield S1 1WB, UK

**Keywords:** cancer stem cells, copper(I) complexes, non-steroidal anti-inflammatory drugs, reactive oxygen species, metallopharmaceuticals

## Abstract

Cancer stem cells (CSCs) are thought to be partly responsible for metastasis and cancer relapse. Currently, there are no effective therapeutic options that can remove CSCs at clinically safe doses. Here, we report the synthesis, characterisation, and anti-breast CSC properties of a series of copper(I) complexes, comprising of non-steroidal anti-inflammatory drugs (NSAIDs) and triphenylphosphine ligands (**1**–**3**). The copper(I) complexes are able to reduce the viability of breast CSCs grown in two- and three-dimensional cultures at micromolar concentrations. The potency of the copper(I) complexes towards breast CSCs was similar to salinomycin (an established anti-breast CSC agent) and cisplatin (a clinically used metallopharmaceutical). Cell-based studies showed that the copper(I) complexes are readily, and similarly, internalised by breast CSCs. The copper(I) complexes significantly increase the intracellular reactive oxygen species (ROS) levels in breast CSCs, and their ROS generation profile with respect to time is dependent on the NSAID component present. The generation of intracellular ROS by the copper(I) complexes could be part of the underlying mechanism by which they evoke breast CSC death. As far as we are aware, this is the first study to explore the anti-breast CSC properties of copper(I) complexes.

## 1. Introduction

Cancer is one of the leading causes of death worldwide, with approximately 10 million deaths in 2020 (which accounted for almost one in six deaths recorded) [1]. The main contributing factors for cancer-related mortalities are metastasis and recurrence [2]. Both metastasis and recurrence are heavily linked to the existence of a hard-to-remove sub-population of tumour cells called cancer stem cells (CSCs) [3]. CSCs make up only a small fraction of a given solid tumour or leukemia population (0.05–3%); however, their presence in biopsies is strongly associated with poor patient outcomes [4,5,6]. CSCs have the capacity for self-renewal and differentiation, which is central to their ability to reform tumours after treatment, and promote the spread of cancer cells from the primary site to other parts of the body [3,7]. Current treatment regimens (such as surgery, chemotherapy, radiation, and immunotherapy) are unable to effectively remove CSCs [8,9]. This is due to a number of complex features intrinsic to CSCs, including their relatively slow cell cycle profile, their high drug efflux properties, and their tendency to reside in inaccessible hypoxic and acidic microenvironments [10]. Over the last few decades, copious amounts of time and money have been invested into developing new anticancer drug candidates that have the ability to remove CSCs at clinically safe doses [8,11]. Many of these efforts have focused on purely organic small molecules; however, we and others have shown that metal complexes can also make effective anti-CSC agents [12,13].

Copper(II) coordination complexes have emerged as a promising class of anti-breast CSC agents [14,15,16,17,18]. Specifically, copper(II) complexes with non-steroidal anti-inflammatory drugs (NSAIDs) are able to selectively or simultaneously kill breast CSCs and bulk breast cancer cells at sub-micromolar and micromolar concentrations [14,15,17]. The copper(II) complexes induce CSC death by elevating reactive oxygen species (ROS) levels as a result of copper(I/II)-associated Fenton-like redox reactions, and by inhibiting cyclooxygenase-2 (COX-2) [14,15,17]. The success of this approach is reliant on the susceptibility of breast CSCs and bulk breast cancer cells to changes in their intracellular redox state, and the overexpression of COX-2 in breast CSCs and bulk breast cancer cells [19,20]. Other classes of copper(II) complexes (with tropolone ligands or bipyridyl and glycinate ligands) have been recently reported to inhibit (breast and prostate) CSC growth at micromolar concentrations; [21,22] however, copper(I) complexes of any type are yet to be explored in this context. Several copper(I) complexes, especially those containing mono- and bi-dentate phosphine ligands with or without additional N-donor ligands, have been reported to display impressive bulk cancer cell potencies (typically in the micromolar range) [23]. According to the relatively large body of work already published on the anti-bulk cancer cell properties of copper(I) complexes, cell toxicity and its associated mechanism of action is highly dependent on the coordinating ligands [24,25,26,27,28,29,30,31]. Four- and two-coordinate copper(I)–phosphine complexes induce a wide range of cellular effects, such as oxidative stress (through the generation of ROS), disruption of genomic DNA function (via intercalation, inhibition of transcription, or cleavage), proteasome inhibition, endoplasmic reticulum stress, vacuolisation, and cell expansion [24,25,26,27,28,29,30,31]. Despite the interest in the anticancer potential of copper(I) complexes, no copper(I) complexes of any geometry have been challenged with CSCs of any tissue type.

**Scheme 1:**
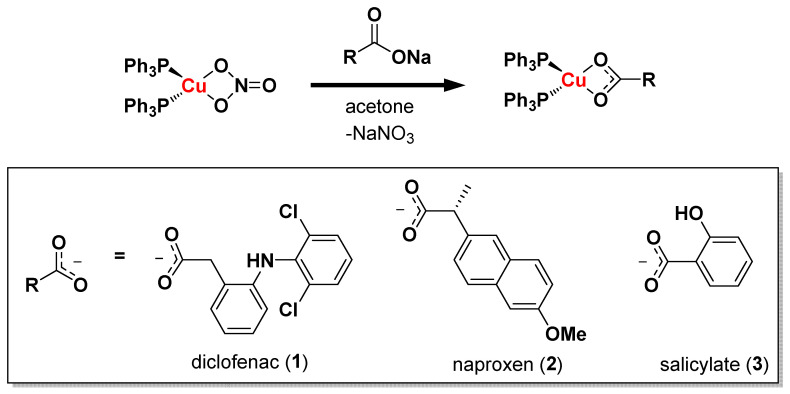
Reaction scheme for the preparation of copper(I) complexes containing triphenylphosphine ligands and diclofenac, naproxen, or salicylate moieties (**1**–**3**).

Here, we have sought to expand the structural scope of anti-breast CSC copper complexes, by developing the series of tetrahedral copper(I)–NSAID complexes **1**–**3** (see Scheme 1 for the chemical structures). The air-stable complexes were prepared via the stabilisation of the copper(I) centre with two bulky triphenylphosphine ligands. Herein, we report the synthesis, characterisation, breast CSC potency, and mechanism of action, in terms of intracellular ROS generation, of the copper(I)–NSAID complexes. To the best of our knowledge, this is the first study to investigate the anti-breast CSC properties of copper(I) complexes of any geometry or ligand composition.

## 2. Results and Discussion

**Figure 1:**
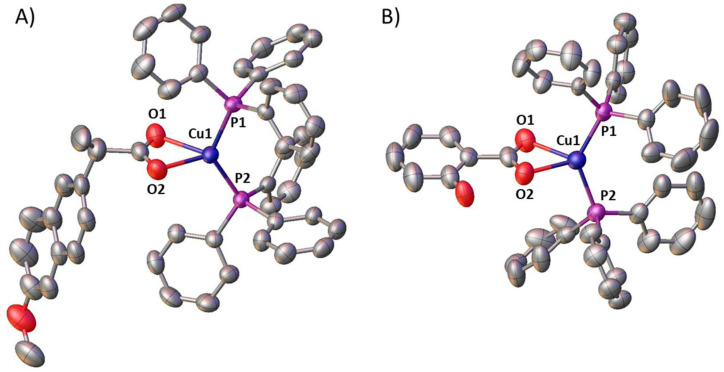
X-ray structures of the copper(I) complexes (**A**) **2** and (**B**) **3**, comprising triphenylphosphine and naproxen or salicylate, respectively. Ellipsoids are shown at 50% probability. C atoms are in grey, P in purple, O in red, and Cu in blue. H atoms and disorder components have been omitted for clarity.

The copper(I)–NSAID complexes **1**–**3** were prepared, as depicted in Scheme 1. Cu(NO_3_)(PPh_3_)_2_ [32] was reacted with an equimolar amount of the corresponding NSAID sodium salt (diclofenac sodium for **1**, naproxen sodium for **2**, and sodium salicylate for **3**) in acetone. Upon the filtration of the resultant solution through celite, concentration, and the addition of hexane, the copper(I)–NSAID complexes **1**–**3** precipitated out, and were isolated as white solids in good yields (65–80%). The copper(I)–NSAID complexes **1**–**3** were fully characterised via ^1^H, ^31^P NMR, and infra-red spectroscopy, high-resolution mass spectrometry, elemental analysis, and single crystal X-ray crystallography (Figures S1–S11, Table 1, Table 2 and Table S1, see the ESI). The expected chemical shifts and multiplicities were observed for the signals in the ^1^H NMR spectra of **1**–**3** (Figures S1, S3, and S5). For **3**, the presence of the hydroxyl signal at 13.07 ppm suggested that the salicylate moiety bound to copper(I) solely via the carboxylate group (Figure S5). The presence and retention of the triphenylphosphine–copper(I) coordination in **1**–**3** was supported by the downfield chemical shift of the ^31^P NMR signal corresponding to **1**–**3** (−2.38 to −3.07 ppm, Figures S2, S4, and S6) compared to free triphenylphosphine (−5.55 ppm, Figure S7). Distinctive molecular ion peaks, corresponding to **1**–**3**, with the appropriate isotopic pattern were observed in the high-resolution ESI-MS (*m*/*z* = 946.0457 [**1**+MeCN+Na-H]^+^; 881.1247 [**2**+MeCN+Na-H]^+^; 789.0626 [**3**+MeCN+Na-H]^+^; Figures S8–S10). The IR spectra for **1**–**3** exhibited asymmetric ν_asym_(CO_2_) and symmetric ν_sym_(CO_2_) carboxylato bands at 1563–1557 cm^−1^ and 1389–1383 cm^−1^, respectively (Figure S11). The difference, Δ, between the ν_asym_(CO_2_) and ν_sym_(CO_2_) stretching bands for **1**–**3** varied between 168 and 180 cm^−1^, indicative of a bidentate coordination mode for the carboxylate group on the NSAIDs to the copper(I) centre (as depicted in Scheme 1) [33,34]. The bulk solid purity of **1**–**3** was established via elemental analysis. Single crystals of **2** and **3,** suitable for X-ray diffraction studies, were obtained via the layer-diffusion of hexane into a DCM solution of **2** and **3** (CCDC 2284762 and 2284763, Figure 1 and Table S1). Selected bond distances and bond angles are presented in Table 1 and Table 2. The structure of **2** and **3** consists of a copper(I) centre with a distorted tetrahedral geometry. The copper(I) centre in **2** and **3** is coordinated to two triphenylphosphine ligands and to the corresponding NSAID via the carboxylate moiety, in a bidentate manner (Figure 1). The copper(I) coordination sphere is consistent with the spectroscopic and analytic data for **2** and **3** described above. Overall, the average Cu-P and Cu-O bond lengths and bond angles around the copper(I) centre observed for **2** and **3** are consistent with the bond parameters reported for related copper(I) complexes. [35,36]

**Figure 2:**
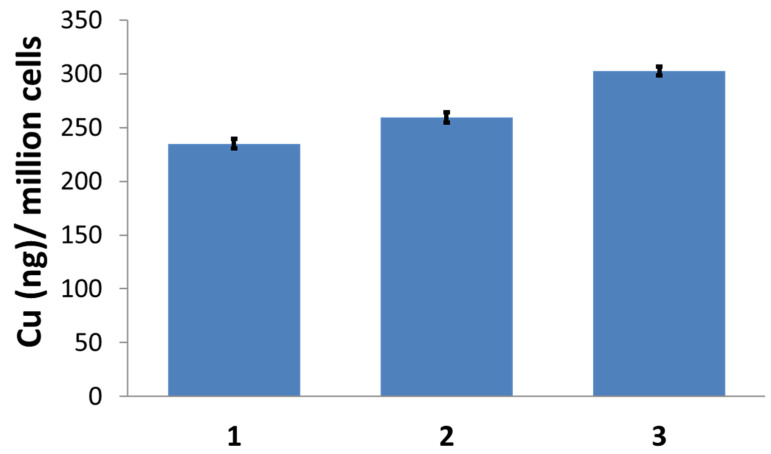
Copper content (ng of Cu/ 10^6^ cells) in HMLER-shEcad cells treated with **1**–**3** (5 µM for 24 h).

Upon the successful synthesis and characterisation of **1**–**3**, their physicochemical properties were investigated. The lipophilicity of **1**–**3** was determined via measuring the ability of **1**–**3** to partition in a mixture of water and octanol (P). The experimentally determined LogP values for **1**–**3** varied between 0.82 ± 0.002 and 1.25 ± 0.06 (Table S2). The hydrophophic nature of **1**–**3** suggests that the copper(I) complexes should have a good bioavailability, and be readily internalised by the dividing cells. Next, the ability of **1**–**3** to be taken up by breast CSCs was determined. HMLER-shEcad cells (which are breast CSC-enriched cells) were incubated with **1**–**3** (5 µM for 24 h), and the copper content in the whole cell was determined via inductively coupled plasma mass spectrometry (ICP-MS). All three copper(I) complexes were taken up by breast CSCs to a good level (taking into account the administration dose): 235 ± 5 ng of Cu/million cells for **1**, 259 ± 5 ng of Cu/ million cells for **2**, and 302 ± 4 ng of Cu/million cells for **3** (Figure 2). The similar breast CSC uptake for **1**–**3** is consistent with their similar LogP values.

The cytotoxicity of the copper(I) complexes **1**–**3** towards bulk breast cancer cells (HMLER) and breast CSCs (HMLER-shEcad) grown in monolayer systems was determined using the colorimetric MTT (3-(4,5-dimethylthiazol-2-yl)-2,5-diphenyltetrazolium bromide) assay. The IC_50_ values (the concentration required to reduce cell viability by 50%) were determined from the dose–response curves (Figures S12–S14), and are reported in Table 3. The copper(I) complexes **1**–**3** exhibited micromolar potency towards bulk breast cancer cells and breast CSCs. There was little variation in the IC_50_ values across the copper(I) complexes towards HMLER and HMLER-shEcad cells, suggesting that the copper(I)-triphenylphosphine component (the common structural motif in **1**–**3**) was the main contributor to their cytotoxicity. This notion is supported by the relatively low potency of the NSAIDs (diclofenac, naproxen, and sodium salicylate) towards HMLER and HMLER-shEcad cells (Figure S15 and Table S3) [15,37]. Notably, the copper(I) complexes **1**–**3** displayed similar potencies as salinomycin (a well-known anti-breast CSC agent) and cisplatin (a clinically applied anticancer agent) towards bulk breast cancer cells and breast CSCs (Table 3) [14,37]. To obtain a sense of the therapeutic potential of **1**–**3**, their cytotoxicity towards non-cancerous bronchial epithelium BEAS-2B cells was determined. The copper(I) complexes **1** (IC_50_ value = 4.32 ± 0.13 µM), **2** (IC_50_ value = 4.11 ± 0.001 µM), and **3** (IC_50_ value = 3.53 ± 0.21 µM) were significantly less active towards BEAS-2B cells than HMLER cells, and similarly active towards HMLER-shEcad cells (Figure S16). Therefore, according to the cytotoxicity studies in monolayer cell systems, **1**–**3** have the potential to preferentially reduce the viability of bulk breast cancer cells over non-cancerous cells at certain micromolar concentrations.

**Figure 3:**
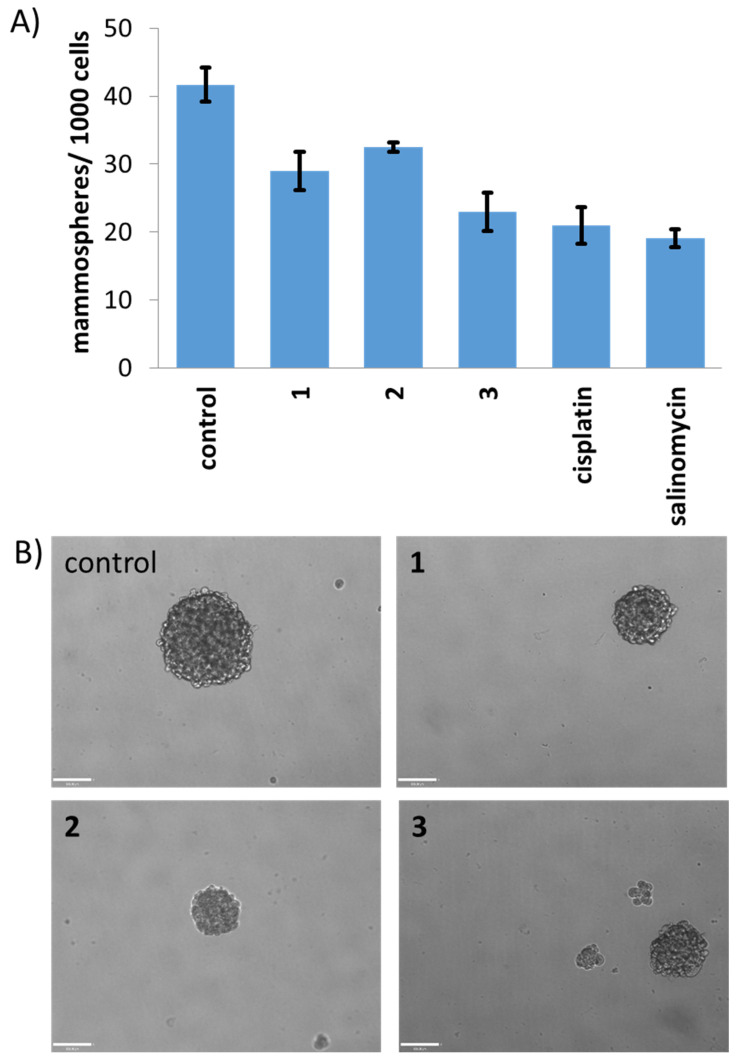
(**A**) Quantification of mammosphere formation with HMLER-shEcad cells, untreated, and treated with **1**–**3**, cisplatin, or salinomycin at 0.5 µM for 5 days. Error bars = SD. (**B**) Representative bright-field images (×10) of the mammospheres, in the absence and presence of **1**–**3**, at 0.5 µM for 5 days.

Mammospheres are three-dimensional structures that form when breast CSCs are cultured in serum-free, low-attachment conditions [38]. Mammospheres provide a reasonable tumour-like model for assessing CSC potency and judging the translatable scope of small-molecule drug candidates. The capacity of the copper(I) complexes **1**–**3** to inhibit mammosphere formation was determined using an inverted microscope. Treatment of single-cell suspensions of HMLER-shEcad cells with **1**–**3** (at 0.5 μM, a non-lethal dose after 5 days of incubation) significantly reduced the number and size of the mammospheres formed (Figure 3A,B). The mammosphere inhibitory effect of **1**–**3** was similar to that observed for cisplatin and salinomycin under identical conditions (Figure 3A) [39,40]. The impact of **1**–**3** on mammosphere viability was determined using TOX8, a resazurin-based reagent that is able to penetrate the three-dimensional architecture of mammospheres, and produce a fluorescent output. The IC_50_ values for **1**–**3** (the dose needed to decrease the viability of mammospheres by one-half) were interpolated from the dose–response curves (Figure S17), and are shown in Table 3. The IC_50_ values for **1**–**3** were in the micromolar range, comparable to the IC_50_ values for salinomycin and cisplatin (Table 3) [39,40]. The mammosphere potency across the copper(I) complexes **1**–**3** based on the IC_50_ values was similar, indicating that the copper(I)-triphenylphosphine component (their shared structural feature) was largely responsible for their anti-mammosphere activity. Taken together, the mammosphere studies show that copper(I) complexes **1**–**3** are able to reduce mammosphere formation, size, and viability to a good level, matching the effect observed for salinomycin and cisplatin.

**Table 3:** The IC_50_ values of the copper(I) complexes **1**–**3**, cisplatin, and salinomycin against HMLER and HMLER-shEcad cells and HMLER-shEcad mammospheres, determined after 72 h or 120 h incubation (mean of two or three independent experiments ± SD).

Compound	HMLER IC_50_ [μM]	HMLER-shEcad IC_50_ [μM]	Mammosphere IC_50_ [μM]
**1**	2.42 ± 0.10	4.61 ± 0.18	18.17 ± 2.02
**2**	2.36 ± 0.03	4.46 ± 0.08	11.80 ± 0.70
**3**	2.13 ± 0.002	4.55 ± 0.001	13.58 ± 0.74
cisplatin ^1^	2.57 ± 0.02	5.65 ± 0.30	13.50 ± 2.34
salinomycin ^1^	11.43 ± 0.42	4.23 ± 0.35	18.50 ± 1.50

^1^ Taken from references [14,37,39,40].

**Figure 4:**
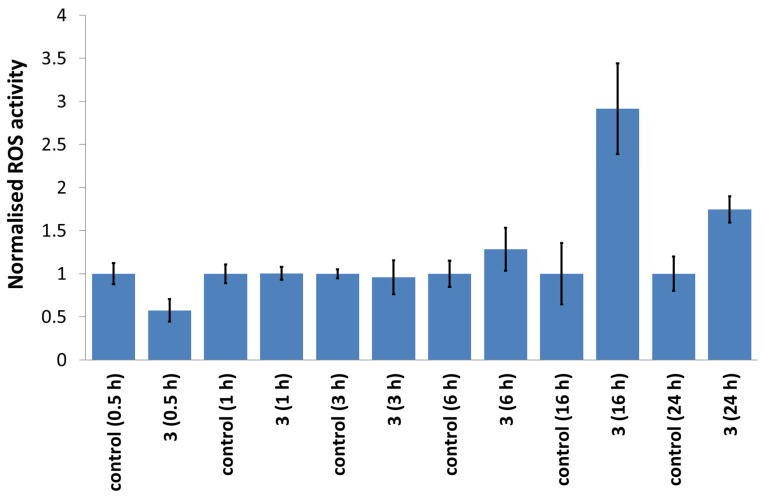
Normalised ROS activity in untreated HMLER-shEcad cells (control) and HMLER-shEcad cells treated with **3** (2 × IC_50_ value, 0.5–24 h).

Copper(I) complexes with phosphine ligands have been reported to promote cell death by increasing intracellular ROS levels [23,41,42]. To determine if the anti-breast CSC activity of **1**–**3** was related to intracellular ROS generation, the ROS levels in HMLER-shEcad cells were measured upon treatment with **1**–**3** (2 × IC_50_ value), using 6-carboxy-2′,7′-dichlorodihydrofluorescein diacetate (DCFH-DA), a well-established ROS indicator. HMLER-shEcad cells exposed to **1** (2 × IC_50_ value) for 1 h exhibited a 76% increase in ROS levels compared to untreated control cells (Figure S18). Exposure to **1** (2 × IC_50_ value) beyond 1 h (3–24 h) did not markedly alter the intracellular ROS status in HMLER-shEcad cells (Figure S18). Treatment of HMLER-shEcad cells with **2** and **3** (2 × IC_50_ value) resulted in large increases in intracellular ROS levels upon prolonged exposure (≥16 h) (Figure 4 and Figure S19). More specifically, **2** increased ROS levels by 41% and 32% after 16 h and 24 h, respectively, while **3** increased ROS levels by 191% and 75% after 16 h and 24 h, respectively (Figure 4 and Figure S19). These results suggest that the NSAID component in **1**–**3** influences their ability to generate intracellular ROS. The diclofenac-bearing copper(I) complex **1** significantly increased ROS levels at a short exposure time (1 h), whereas the naproxen- and salicylate-bearing copper(I) complexes **2** and **3** increased ROS levels at longer exposure times (16 h and 24 h). Notably, the salicylate-containing copper(I) complex **3** increased ROS levels to the greatest extent within the series (191% increase after 16 h exposure, Figure 4). Overall, the ROS studies suggests that the mechanism of action by which **1**–**3** induce breast CSC death could be associated with their ability to generate intracellular ROS.

## 3. Materials and Methods 

### 3.1. Spectroscopic and Analytical Methods

All synthetic procedures were performed under normal atmospheric conditions. ^1^H and ^31^P{^1^H} NMR were recorded at room temperature on a Bruker Avance 400 spectrometer (^1^H 400.0 MHz, ^31^P 162.0 MHz) with chemical shifts (δ, ppm) reported relative to the solvent peaks of the deuterated solvent. Fourier transform infrared (FTIR) spectra were recorded with an IRAffinity-1S Shimadzu spectrophotometer. UV–Vis absorption spectra were recorded on a Cary 3500 UV–Vis spectrophotometer. ICP-MS was performed using a Thermo Scientific, Waltham, MA, USA, ICAP-Qc quadrupole ICP mass spectrometer. Elemental analysis of the compounds prepared was performed commercially by the University of Cambridge. Cu(NO_3_)(PPh_3_)_2_ was prepared, using a previously reported protocol [32]. Sodium salts of the NSAIDs (naproxen, diclofenac, and salicylic acid) were purchased from Sigma-Aldrich, Burlingthon, MA, USA, and used without further purification. Solvents were purchased from Fisher, and used without further purification.

### 3.2. Synthesis of [Cu^I^(diclofenac)(PPh_3_)_2_] (***1***)

To a solution of sodium diclofenac (64 mg, 0.2 mmol) in acetone (5 mL), we added Cu(NO_3_)(PPh_3_)_2_ (130 mg, 0.2 mmol). The resultant mixture was stirred for 1 h. The solution was then filtered through celite, and concentrated to minimum volume under reduced pressure. Then, hexane (20 mL) was added, leading to a white precipitate. The precipitate was collected via filtration, to yield **1** as a white solid (121 mg, 68%). Its properties are: ^1^H NMR (400 MHz, CD_2_Cl_2_) δ 8.89 (s, 1H), 7.38–7.28 (m, 20H), 7.23–7.19 (m, 13H), 7.09 (t, 1H), 6.97–6.89 (m, 2H), 6.46 (d, 1H), 3.73 (s, 2H); ^31^P NMR (162 MHz, CD_2_Cl_2_) δ -2.99 (s, PPh_3_); ATR-FTIR (solid, cm^−1^): 3215, 3053, 1571, 1557, 1510, 1476, 1453, 1433, 1389, 1308, 1093, 763, 744, 691, 648, 529, 501, 495, 438; HR ESI-MS calcd. for C_50_H_40_Cl_2_CuNO_2_P_2_+MeCN+Na-H [M+MeCN+Na-H]^+^: 946.1279, found [M+MeCN+Na-H]^+^: 946.0457; anal. calcd. C_50_H_40_Cl_2_CuNO_2_P_2_·1.5H_2_O: C 65.97, H 4.76, N 1.54, found: C 65.74, H 4.28, N 1.49. 

### 3.3. Synthesis of [Cu^I^(naproxen)(PPh_3_)_2_] (***2***)

To a solution of naproxen sodium (50 mg, 0.2 mmol) in acetone (5 mL), we added Cu(NO_3_)(PPh_3_)_2_ (130 mg, 0.2 mmol). The resultant mixture was stirred for 1 h. The solution was then filtered through celite, and concentrated to minimum volume under reduced pressure. Then, hexane (20 mL) was added, leading to a white precipitate. The precipitate was collected via filtration, to yield **2** as a white solid (130 mg, 80%). Its properties are: ^1^H NMR (400 MHz, CD_2_Cl_2_) δ 7.74 (s, 1H), 7.67–7.64 (m, 2H), 7.55 (dd, 1H), 7.38–7.31 (m, 18H), 7.22–7.17 (m, 13H), 7.12 (dd, 1H), 3.95 (s, 3H) 3.80 (q, 1H), 1.54 (d, 3H); ^31^P NMR (162 MHz, CD_2_Cl_2_) δ -3.07 (s, PPh_3_); ATR-FTIR (solid, cm^−1^): 3052, 1587, 1563, 1480, 1460, 1433, 1383, 1356, 1306, 1252, 1090, 862, 813, 741, 690, 665, 504, 490, 429, 391; HR ESI-MS calcd. For C_50_H_43_CuO_3_P_2_+MeCN+Na-H [M+MeCN+Na-H]^+^: 881.2073, found [M+MeCN+Na-H]^+^: 881.1247; anal. calcd. C_50_H_43_CuO_3_P_2_: C 73.47, H 5.30, N 0.00, found: C 73.47, H 5.26, N 0.00. 

### 3.4. Synthesis of [Cu^I^(salicylate)(PPh_3_)_2_] (***3***)

To a solution of sodium salicylate (32 mg, 0.2 mmol) in acetone (5 mL), we added Cu(NO_3_)(PPh_3_)_2_ (130 mg, 0.2 mmol). The resultant mixture was stirred for 1 h. The solution was then filtered through celite, and concentrated to minimum volume under reduced pressure. Then, hexane (20 mL) was added, leading to a white precipitate. The precipitate was collected via filtration, to yield **3** as a white solid (94 mg, 65%). Its properties are: ^1^H NMR (400 MHz, CD_2_Cl_2_) δ 13.07 (s, 1H), 7.95 (dd, 1H), 7.43–7.39 (m, 18H), 7.34–7.27 (m, 13H), 6.86–6.79 (m, 2H); ^31^P NMR (162 MHz, CD_2_Cl_2_) δ -2.38 (s, PPh_3_); ATR-FTIR (solid, cm^−1^): 3058, 2951, 2923, 1603, 1557, 1478, 1434, 1387, 1262, 1227, 1211, 1154, 1093, 1027, 999, 924, 881, 874, 806, 745, 692, 514, 498, 431; HR ESI-MS calcd. for C_43_H_35_CuO_3_P_2_+MeCN+Na-H [M+MeCN+Na-H]^+^: 789.1443, found [M+MeCN+Na-H]^+^: 789.0626; anal. calcd. C_43_H_35_CuO_3_P_2_·H_2_O: C 69.49, H 5.02, N 0.00, found: C 69.52, H 4.49, N 0.00.

### 3.5. X-ray Crystallography

Crystals of **2** and **3** were mounted in inert oil on glass fibers, and transferred to a Bruker Apex 2000 CCD area detector diffractometer. Data were collected using graphite-monochromated Mo-Kα radiation (λ = 0.71073) at 150(2) K, scan type ϖ. Absorption corrections based on multiple scans were applied, using SADABS [43] or spherical harmonics implemented in the SCALE3 ABSPACK scaling algorithm [44]. The structures were solved via direct methods, and refined on *F^2^*, using the program SHELXT-2016 [45]. Hydrogen atoms were placed at calculated positions, and refined using a riding model, whilst all non-hydrogen atoms were refined anisotropically. The CCDC deposition numbers 2284762 and 2284763 contain the supplementary crystallographic data. These data can be obtained free of charge via the Cambridge Crystallography Data Centre. In the case of **2**, one of the methyl groups of the NSAID ligand (C49) displayed positional disorder. This was modelled over two positions, and the two components refined competitively, converging at a ratio of 0.507(8):0.493(8). Similarity restraints were applied to the anisotropic displacement parameters of atoms C38, C49A, and C49B. In the case of **3**, the OH group of the carboxylate ligand (O3) displayed positional disorder, with its position partially occupied by a H atom; this disorder is complementary to the other *ortho* position on the same ring. This was modelled over two parts, and the two components refined competitively, converging at a ratio of 0.761(5):0.239(5). Chemically equivalent C–O distances were restrained, to be approximately equal.

### 3.6. Measurement of Water–Octanol Partition Coefficient (LogP)

The LogP value for **1**–**3** was determined, using the shake-flask method and ICP-MS. The 1-octanol used in this experiment was pre-saturated with water. A DMSO solution of **1**–**3** (10 μL, 10 mM) was incubated with 1-octanol (495 μL) and H_2_O (495 μL) in a 1.5 mL tube. The tube was shaken at room temperature for 24 h. The two phases were separated via centrifugation, and the copper content in the water phase was determined via ICP-MS.

### 3.7. Cell Culture

The human mammary epithelial cell lines, HMLER and HMLER-shEcad were kindly donated by Prof. R. A. Weinberg (Whitehead Institute, MIT). HMLER and HMLER-shEcad cells were maintained in mammary epithelial cell growth medium (MEGM) with supplements and growth factors (BPE, hydrocortisone, hEGF, insulin, and gentamicin/amphotericin-B). The BEAS-2B bronchial epithelium cell line was acquired from the American Type Culture Collection (ATCC, Manassas, VA, USA), and cultured in RPMI 1640 medium, with 2 mM L-glutamine supplemented with 1% penicillin and 10% fetal bovine serum. The cells were grown at 310 K in a humidified atmosphere containing 5% CO_2_.

### 3.8. Antiproliferative Studies: MTT Assay

Exponentially growing cells were seeded at a density of approximately 5 × 10^3^ cells per well in 96-well flat-bottomed microplates, and allowed to attach for 24 h prior to the addition of compounds. Various concentrations of the test compounds (0.19–100 μM) were added, and the plates were incubated for 72 h at 37 °C (total volume 200 μL). Stock solutions of the compounds were prepared as 10 mM DMSO solutions, and diluted using cell media. The final concentration of DMSO in each well was ≤1 %. After 72 h, 20 μL of MTT (4 mg mL^−1^ in PBS) was added to each well, and the plates were incubated for an additional 4 h at 37 °C. The media/MTT mixture was eliminated, and DMSO (100 μL per well) was added to dissolve the formazan precipitates. The optical density was measured at 550 nm, using a 96-well multiscanner autoreader. Absorbance values were normalised to (DMSO-containing) control wells, and plotted as concentration of compound versus % cell viability. IC_50_ values were interpolated from the resulting dose-dependent curves. The reported IC_50_ values are the average of three independent experiments (n = 18).

### 3.9. Mammosphere Formation and Viability Assay

HMLER-shEcad cells (5 × 10^3^) were plated in ultralow-attachment 96-well plates (Corning) and incubated in MEGM supplemented with B27 (Invitrogen), 20 ng mL^−1^ EGF, and 4 μg mL^−1^ heparin (Sigma), for 5 days. Studies were also conducted in the presence of **1**–**3**, cisplatin and salinomycin (0–133 µM). Mammospheres treated with **1**–**3**, cisplatin, and salinomycin (0.5 µM, 5 days) were counted and imaged, using an inverted microscope. The viability of the mammospheres was determined via the addition of a resazurin-based reagent, TOX8 (Sigma). After incubation for 16 h, the fluorescence of the solutions was read at 590 nm (λ_ex_ = 560 nm). Viable mammospheres reduce the amount of the oxidised TOX8 form (blue) and, concurrently, increase the amount of the fluorescent TOX8 intermediate (red), indicating the degree of mammosphere cytotoxicity caused by the test compound. Fluorescence values were normalised to DMSO-containing controls, and plotted as concentration of test compound versus % mammospheres viability. IC_50_ values were interpolated from the resulting dose-dependent curves. The reported IC_50_ values are the average of two independent experiments, each consisting of two replicates per concentration level (n = 4).

### 3.10. Cellular Uptake

To measure the cellular uptake of **1**–**3**, about 1 million HMLER-shEcad cells were treated with **1**–**3** (5 μM) at 37 °C for 24 h. After incubation, the media were removed, and the cells were washed with PBS (2 mL × 3), and harvested. The number of cells was counted at this stage, using a haemocytometer. This mitigates any cell death induced by **1**–**3** at the administered concentration, and experimental cell loss. The cellular pellet was dissolved in 65% HNO_3_ (250 µL) overnight. All samples were diluted 17-fold with water, and analysed using inductively coupled plasma mass spectrometry (ICP-MS, using the Thermo Scientific ICAP-Qc quadrupole ICP mass spectrometer). Copper levels are expressed as mass of Cu (ng) per million cells. Results are presented as the mean of four determinations for each data point.

### 3.11. Intracellular ROS Assay

HMLER-shEcad cells (5 × 10^3^) were seeded in each well of a 96-well plate. After the cells were incubated overnight, they were treated with **1**–**3** (2 × IC_50_ value, 0.5–24 h), and incubated with 6-carboxy-2′,7′-dichlorodihydrofluorescein diacetate (20 μM) for 90 min. The intracellular ROS level was determined via measurement of the fluorescence of the solutions in each well at 529 nm (λ_ex_ = 504 nm).

## 4. Conclusions

In summary, we report the preparation, characterisation, and anti-breast CSC properties of the series of copper(I) complexes **1**–**3**, comprising two triphenylphosphine ligands and a NSAID (diclofenac, naproxen or salicylate). The NSAID moiety was shown to bind to the copper(I) centre in a bidentate fashion via both carboxylate oxygen atoms. NMR and IR spectroscopy and mass spectrometry studies were conducted to prove the structural composition of **1**–**3**. Furthermore, single-crystal X-ray crystallography studies unambiguously showed that **2** and **3** adopt distorted tetrahedral geometries, which is the most common conformation for four-coordinate copper(I) complexes. The copper(I) complexes **1**–**3** were relatively hydrophobic (LogP values = 0.82–1.25) and, hence, were readily taken up by breast CSCs (235–302 ng of Cu/million cells upon dosage at 5 µM for 24 h). The copper(I) complexes **1**–**3** displayed micromolar toxicity towards breast CSCs grown in the monolayer and three-dimensional (mammosphere) systems. This was comparable to the potency reported for salinomycin and cisplatin under identical conditions. The copper(I) complexes **1**–**3** were able to significantly increase intracellular ROS levels in breast CSCs at different time points across a 24 h window, dependent on the NSAID motif present. The diclofenac-bearing copper(I) complex **1** induced a significant increase in intracellular ROS levels at a short exposure time (1 h), while the naproxen- and diclofenac-bearing copper(I) complexes **2** and **3** induced a significant increase in intracellular ROS levels at longer exposure times (16 h and 24 h). The mechanism of breast CSC toxicity of **1**–**3** could be related, in part, to their ability to generate intracellular ROS. In this study we report the anti-breast CSC potential of copper(I) complexes for the first time, and expand the therapeutic scope of metal–NSAID complexes. Further, we believe that this work opens the door for the development of other copper(I) complexes as anti-CSC agents.

## Figures and Tables

**Table 1:** Selected bond lengths (Å) and angles (°) for complex **2**.

Cu(1)-O(2)	2.188(3)	Cu(1)-O(1)	2.238(3)
Cu(1)-P(2)	2.2305(13)	Cu(1)-P(1)	2.2513(11)
O(2)-Cu(1)-P(2)	118.00(8)	O(2)-Cu(1)-P(1)	108.05(8)
O(2)-Cu(1)-O(1)	58.99(10)	P(2)-Cu(1)-P(1)	128.52(4)
P(2)-Cu(1)-O(1)	117.57(8)	O(1)-Cu(1)-P(1)	104.84(8)

**Table 2:** Selected bond lengths (Å) and angles (°) for complex **3**.

Cu(1)-O(2)	2.174(3)	Cu(1)-O(1)	2.298(3)
Cu(1)-P(2)	2.2318(13)	Cu(1)-P(1)	2.2210(13)
O(2)-Cu(1)-P(2)	102.56(7)	O(2)-Cu(1)-P(1)	118.41(7)
O(2)-Cu(1)-O(1)	58.73(9)	P(2)-Cu(1)-P(1)	133.16(5)
P(2)-Cu(1)-O(1)	114.54(7)	O(1)-Cu(1)-P(1)	106.02(7)

## Data Availability

Not applicable.

## Supplementary Materials

The following supporting information can be downloaded at: https://www.mdpi.com/article/10.3390/molecules28176401/s1.

## Sample Availability

Samples of the compounds are available from the authors upon request.
