# Supplementary material for: The Anti-Breast Cancer Stem Cell Potency of Copper(I)-Non-Steroidal Anti-Inflammatory Drug Complexes
Source: Molecules. 2023 Sep 1;28(17):6401. doi: 10.3390/molecules28176401 (PMC10489748; doi:10.3390/molecules28176401)
Supplement: Supplementary file 1 [file molecules-28-06401-s001.zip › molecules-2575413-supplementary.pdf]

Supporting Information for

The anti-breast cancer stem cell potency of copper(I)-non-steroidal anti-inflammatory drugs complexes

Alice Johnson [1,2,†], Xiao Feng [1,†], Kuldip Singh [1], Fabrizio Ortu [1,*] and Kogularamanan Suntharalingam [1,*]

[1] School of Chemistry, University of Leicester, Leicester LE1 7RH, UK; alice.johnson@shu.ac.uk (A.J.); xf40@leicester.ac.uk (X.F.); ks42@leicester.ac.uk (K.S.)

[2] Biomolecular Sciences Research Centre, Sheffield Hallam University, Sheffield S1 1WB, UK

* Correspondence: fabrizio.ortu@leicester.ac.uk (F.O.); k.suntharalingam@leicester.ac.uk (K.S.); Tel.: +44-(0)116-294-4670 (F.O.); +44-(0)116-294-4562 (K.S.)

† These authors contributed equally to this work.

Table of Content

Figure S1. ^{1}H NMR spectrum of **1** in CD_2Cl_2.
Figure S2. ^{31}P{^{1}H} NMR spectrum of **1** in CD_2Cl_2.
Figure S3. ^{1}H NMR spectrum of **2** in CD_2Cl_2.
Figure S4. ^{31}P{^{1}H} NMR spectrum of **2** in CD_2Cl_2.
Figure S5. ^{1}H NMR spectrum of **3** in CD_2Cl_2.
Figure S6. ^{31}P{^{1}H} NMR spectrum of **3** in CD_2Cl_2.
Figure S7. ^{31}P{^{1}H} NMR spectrum of free triphenylphosphine in CD_2Cl_2.
Figure S8. High resolution ESI mass spectrum (positive mode) of **1**.
Figure S9. High resolution ESI mass spectrum (positive mode) of **2**.
Figure S10. High resolution ESI mass spectrum (positive mode) of **3**.
Figure S11. ATR-FTIR spectra of **1-3** (A-C) in the solid form.
Table S1. Crystallographic data for complexes **2** and **3**.
Table S2. Experimentally determined LogP values for **1-3**.
Figure S12. Representative dose-response curves for the treatment of HMLER and HMLER-shEcad cells with **1** after 72 h incubation.
Figure S13. Representative dose-response curves for the treatment of HMLER and HMLER-shEcad cells with **2** after 72 h incubation.
Figure S14. Representative dose-response curves for the treatment of HMLER and HMLER-shEcad cells with **3** after 72 h incubation.
Figure S15. Representative dose-response curves for the treatment of HMLER and HMLER-shEcad cells with sodium salicylate after 72 h incubation.
Table S3. IC_{50} values of diclofenac, naproxen, and sodium salicylate against HMLER and HMLER-shEcad cells. [1]Determined after 72 h incubation (mean of three independent experiments ± SD).

Figure S16. Representative dose-response curves for the treatment of BEAS-2B cells with **1-3** after 72 h incubation.

Figure S17. Representative dose-response curves for the treatment of HMLER-shEcad mammospheres with **1-3** after 5 days incubation.

Figure S18. Normalised ROS activity in untreated HMLER-shEcad cells (control) and HMLER-shEcad cells treated with **1** (2 × IC_{50} value, 0.5-24 h).

Figure S19. Normalised ROS activity in untreated HMLER-shEcad cells (control) and HMLER-shEcad cells treated with **2** (2 × IC_{50} value, 0.5-24 h).

References

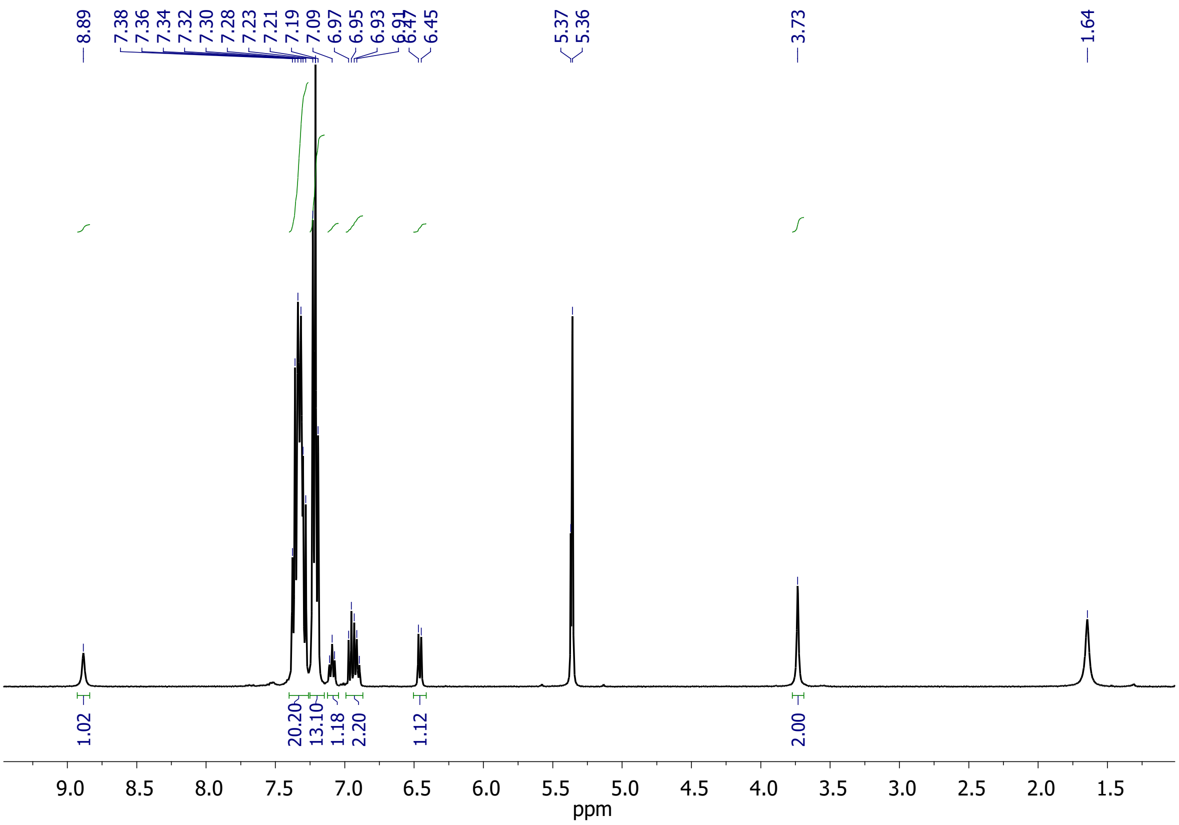

Figure S1. ^{1}H NMR spectrum of **1** in CD_2Cl_2.

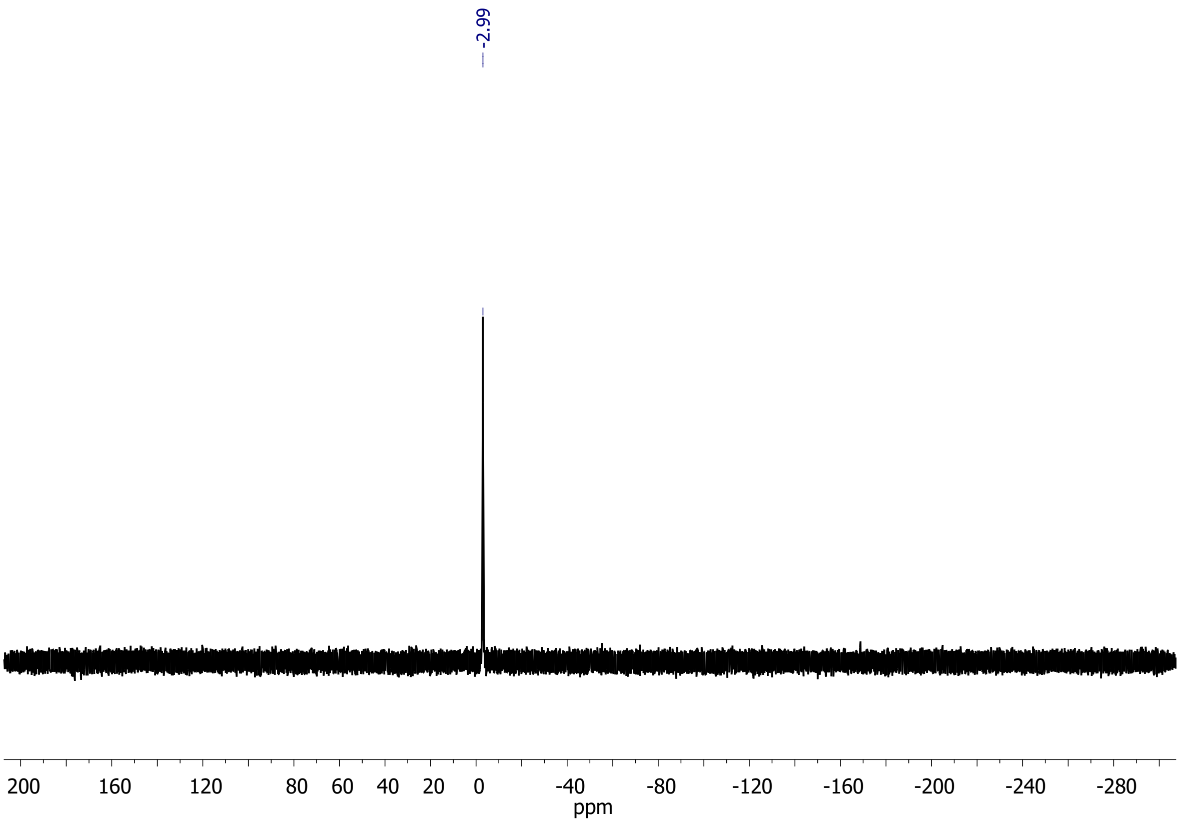

Figure S2. $^{31}P\{^{1}H\}$ NMR spectrum of **1** in CD_2Cl_2.

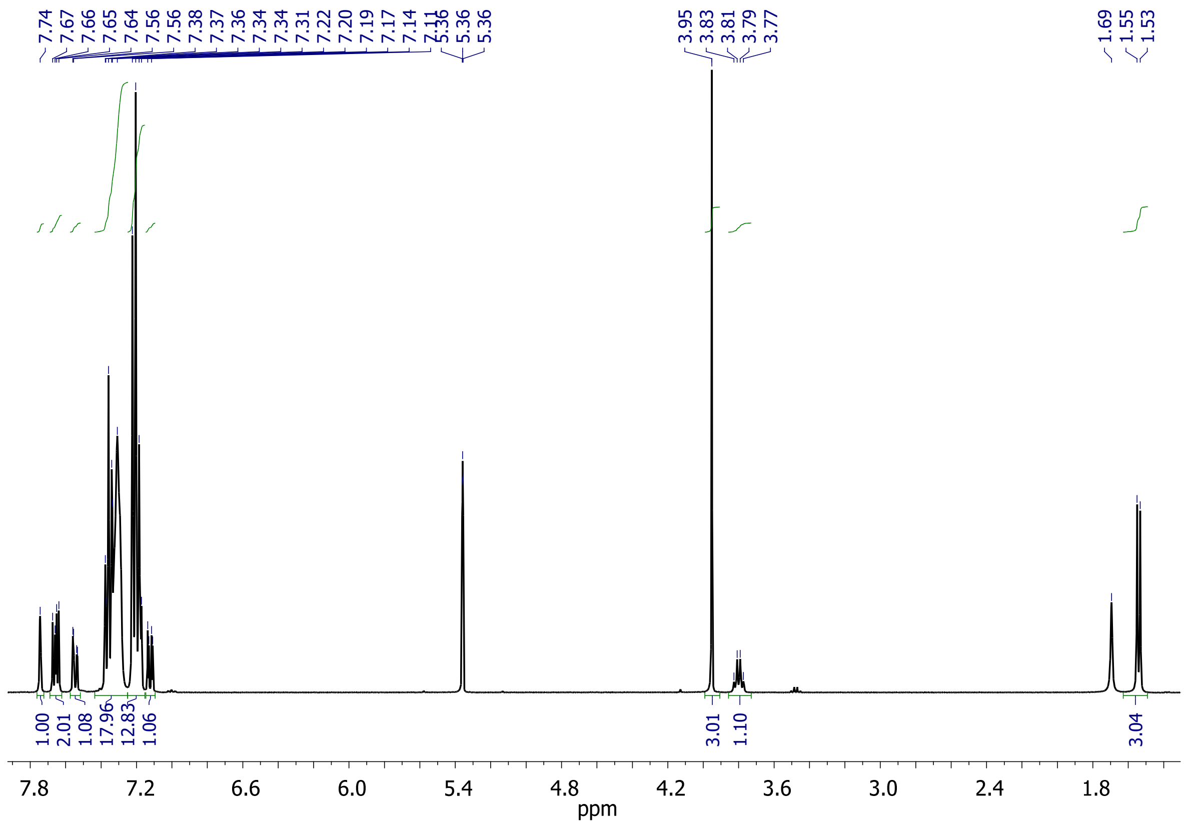

Figure S3. ^{1}H NMR spectrum of **2** in CD_2Cl_2.

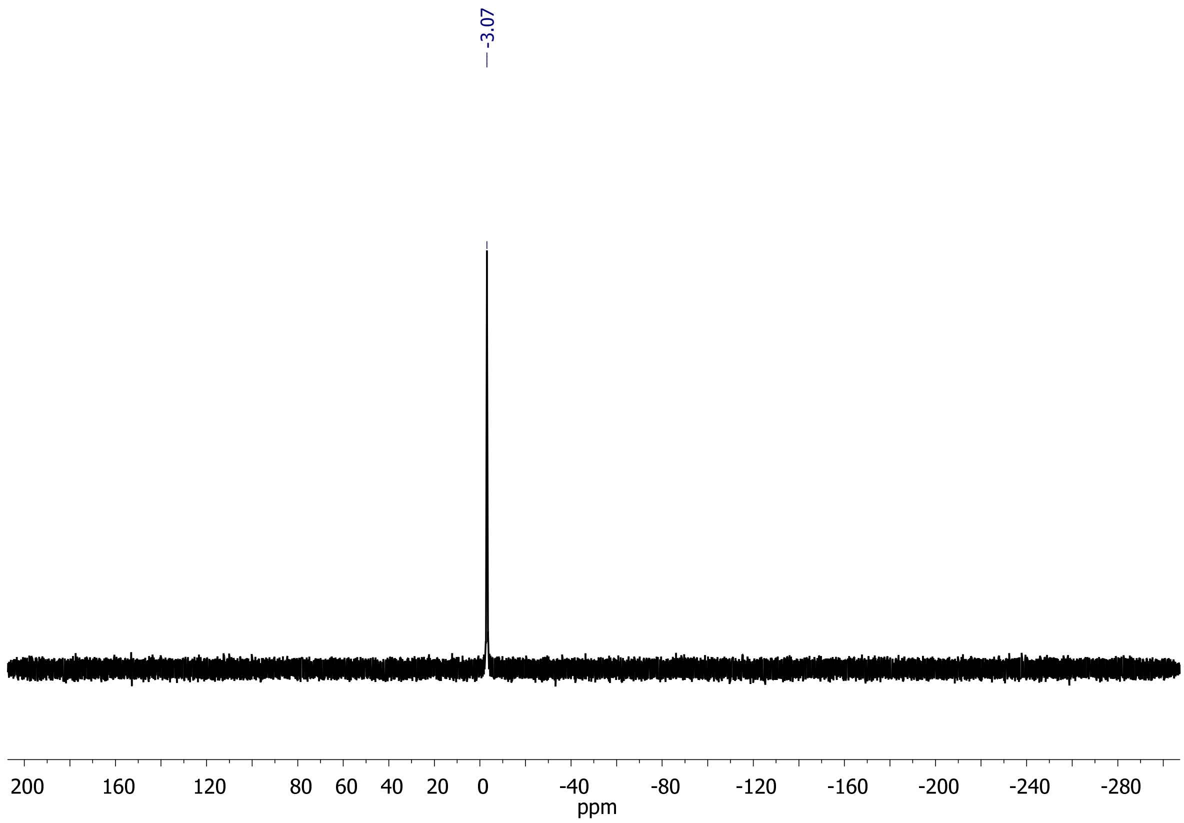

Figure S4. $^{31}P\{^{1}H\}$ NMR spectrum of **2** in CD_2Cl_2.

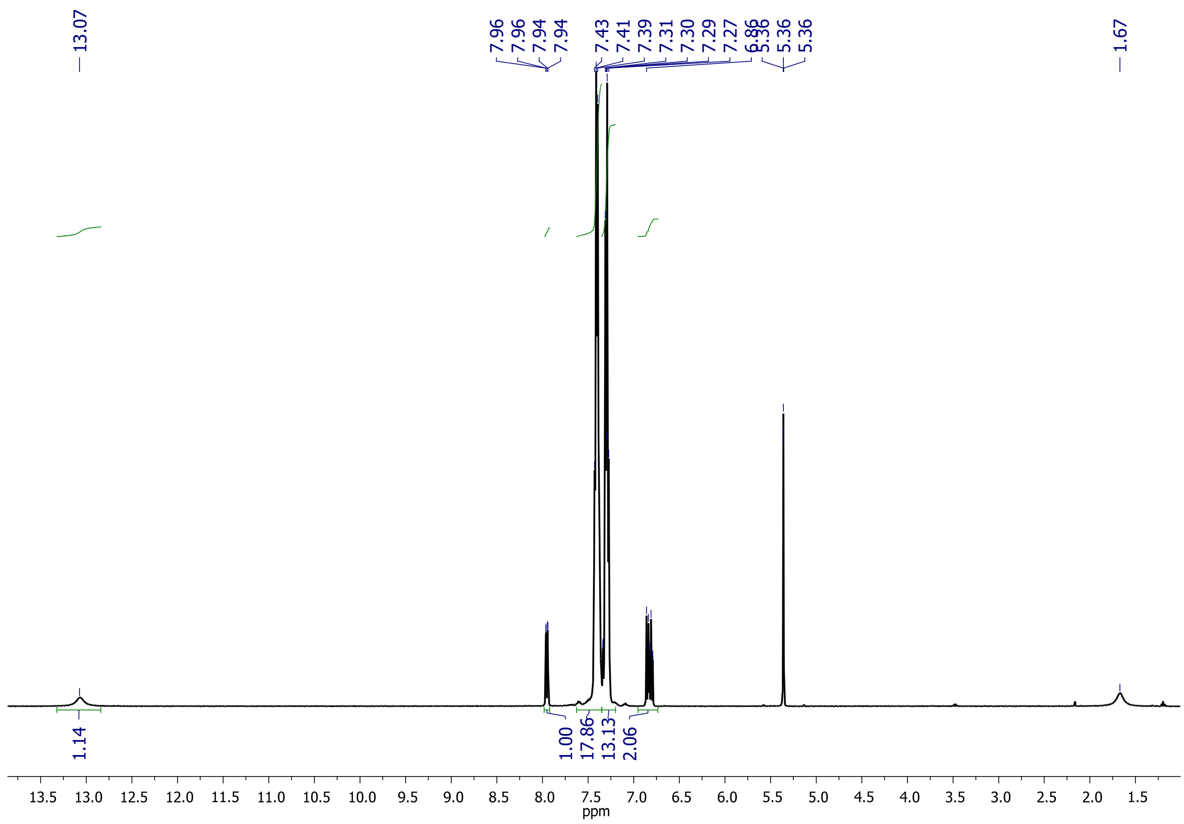

Figure S5. ^{1}H NMR spectrum of **3** in CD_2Cl_2.

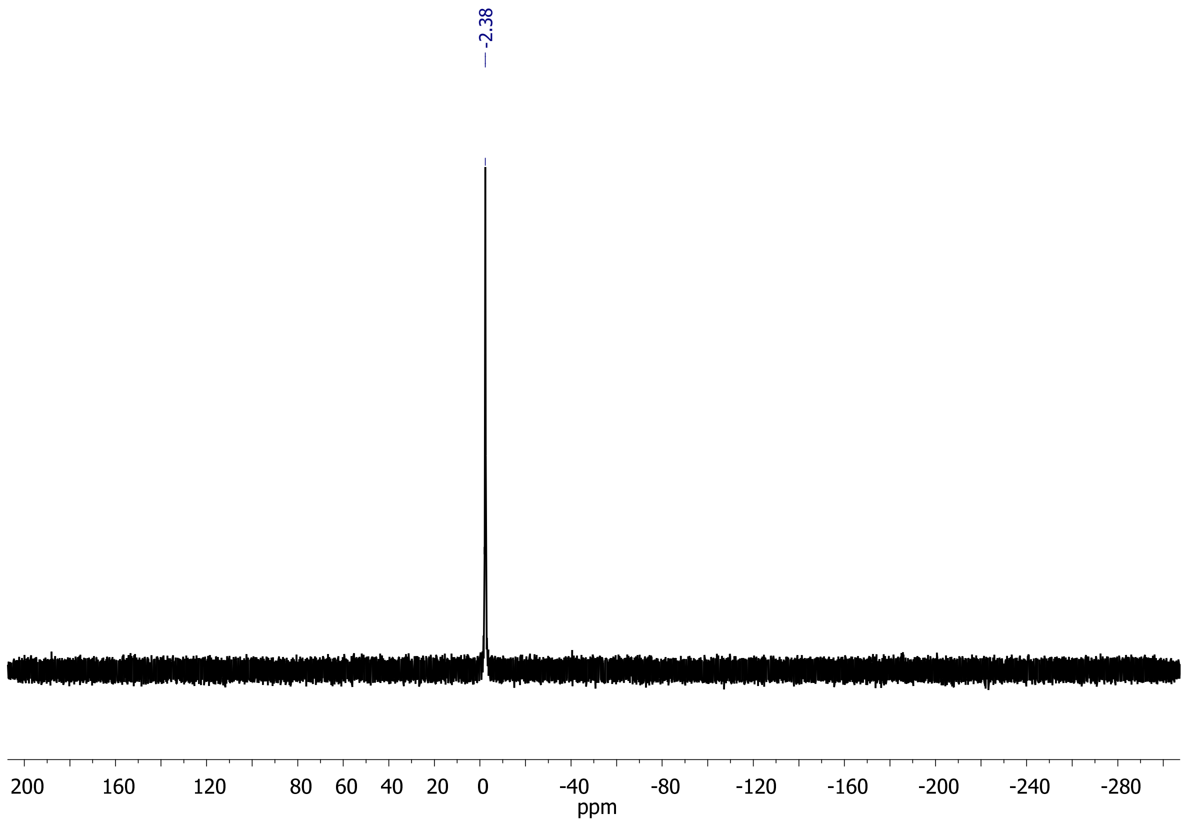

Figure S6. $^{31}P\{^{1}H\}$ NMR spectrum of **3** in CD_2Cl_2.

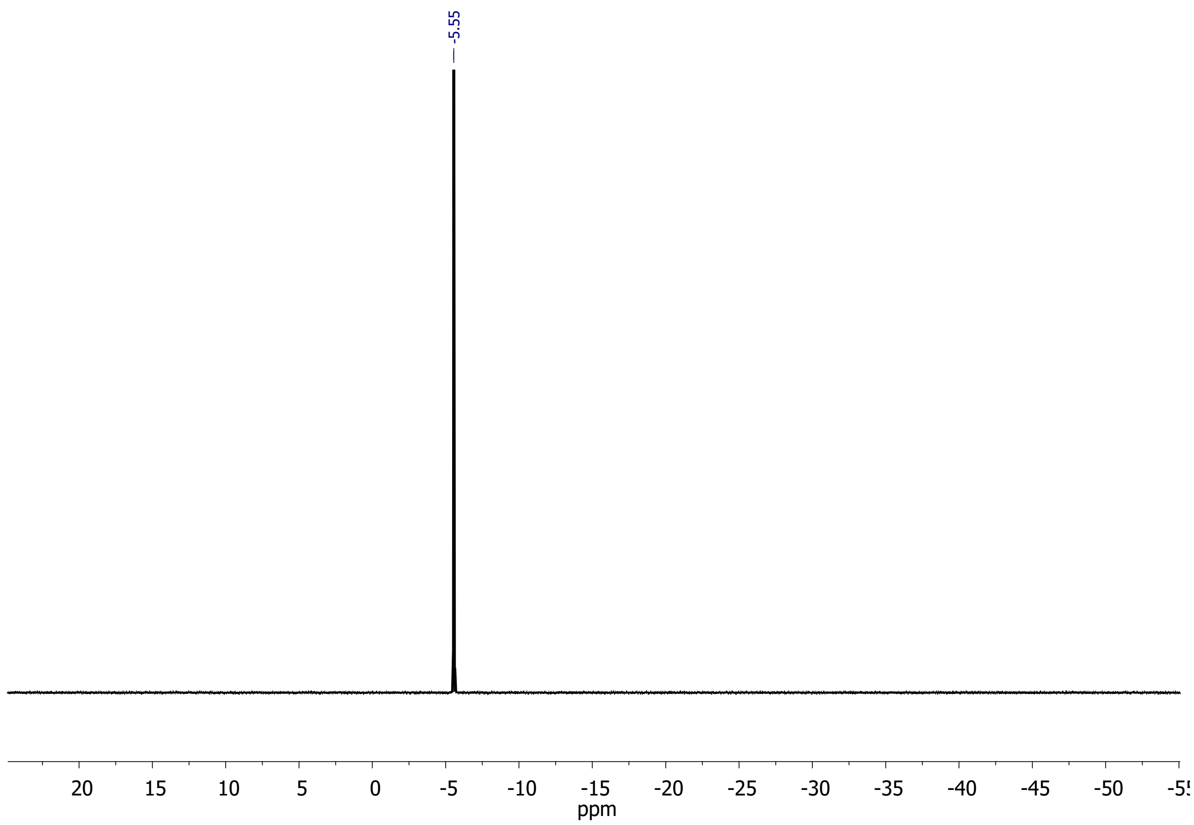

Figure S7. $^{31}P\{^{1}H\}$ NMR spectrum of free triphenylphosphine in CD_2Cl_2.

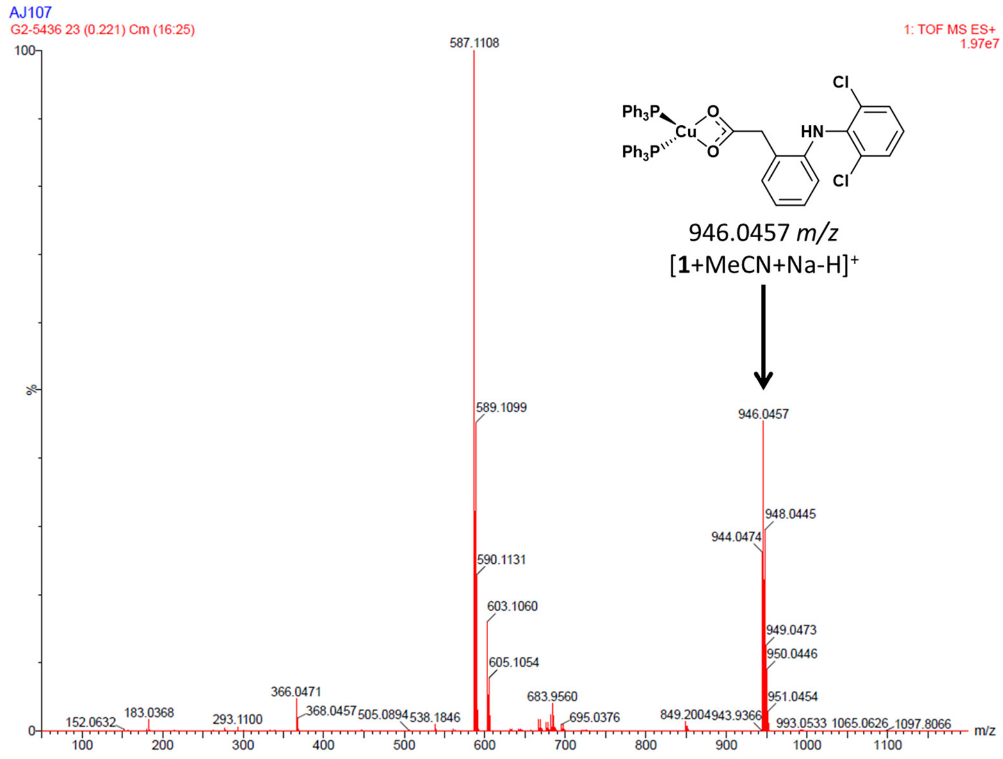

Figure S8. High resolution ESI mass spectrum (positive mode) of **1**.

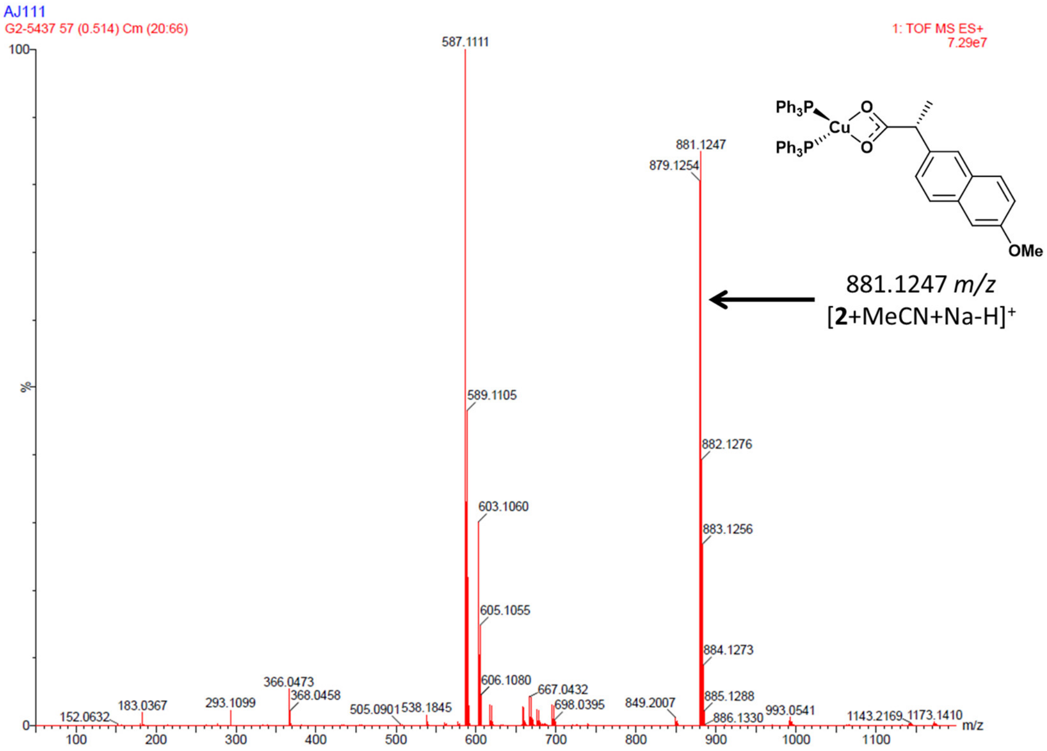

Figure S9. High resolution ESI mass spectrum (positive mode) of **2**.

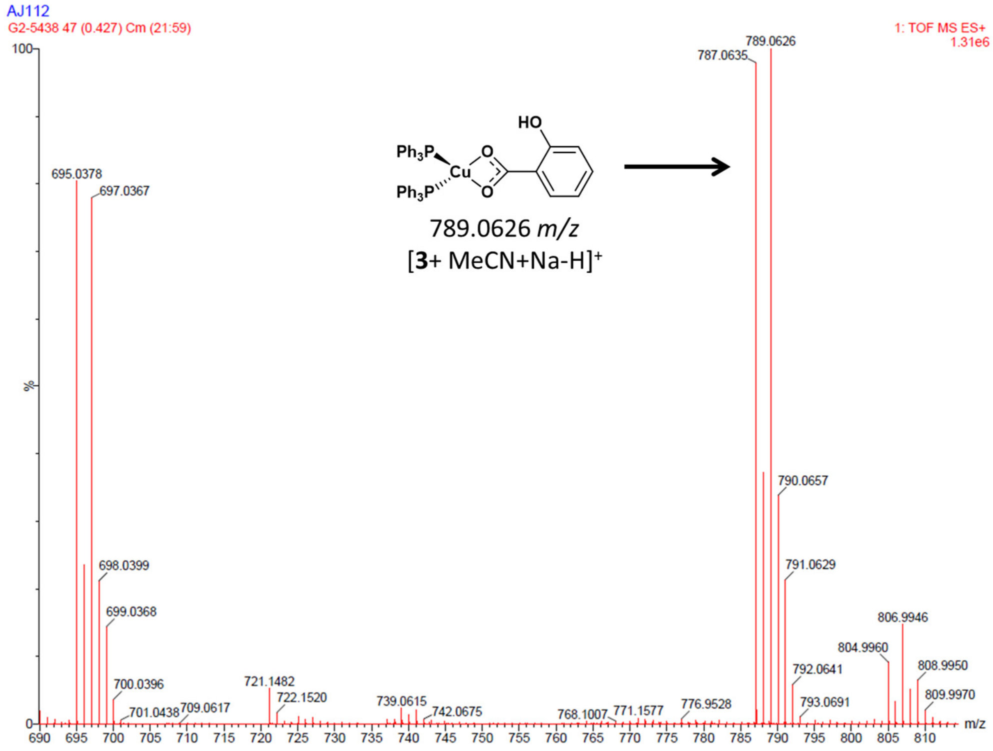

Figure S10. High resolution ESI mass spectrum (positive mode) of **3**.

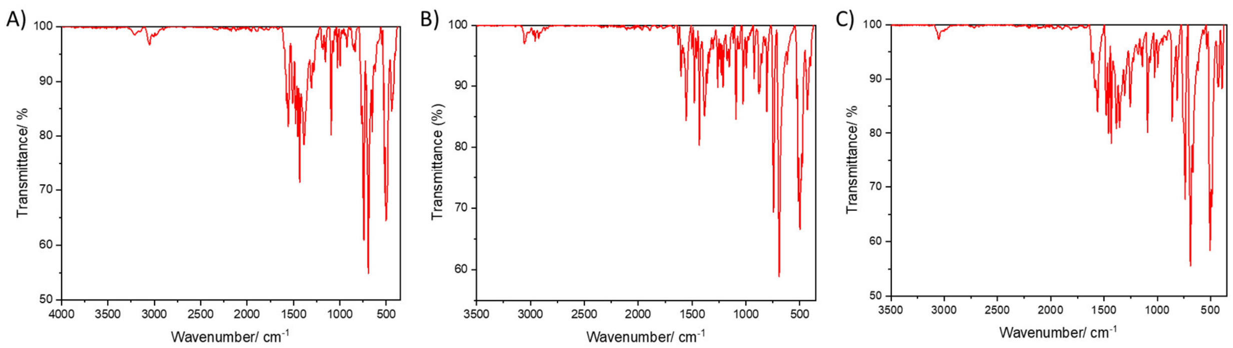

Figure S11. ATR-FTIR spectra of **1-3** (A-C) in the solid form.

Table S1. Crystallographic data for complexes **2** and **3**.

**Metal complex**	**2**	**3**
CCDC No.	2284762	2284763
formula	$C_{50}H_{43}CuO_3P_2$	$C_{43}H_{35}CuO_3P_2$
Fw	817.32	725.19
Crystal system	Triclinic	Monoclinic
Space group	*P*-1	$P2_1/c$
a, Å	12.432(5)	15.354(5)
b, Å	12.788(5)	14.482(5)
c, Å	15.882(7)	17.618(6)
α, deg.	71.055(7)	90
β, deg.	87.972(8)	113.502(6)
γ, deg.	62.694(7)	90
V, Å^3	2102.5(15)	3593(2)
Z	2	4
D_{calcd}, Mg/m^3	1.291	1.341
2θ / deg.	2.736 to 51.998	2.892 to 51.994
Reflections collected	16442	27590
Independent reflections	8144	7043
Goodness-of-fit on F^2	0.959	0.852
R_1, wR_2 [$I \geq 2\sigma(I)$]	0.0574, 0.1164	0.0579, 0.1016
R_1, wR_2 [all data]	0.0930, 0.1268	0.1366, 0.1205

Table S2. Experimentally determined LogP values for **1-3**.

Copper(I) complex	LogP
**1**	1.25 ± 0.06
**2**	1.00 ± 0.09
**3**	0.82 ± 0.002

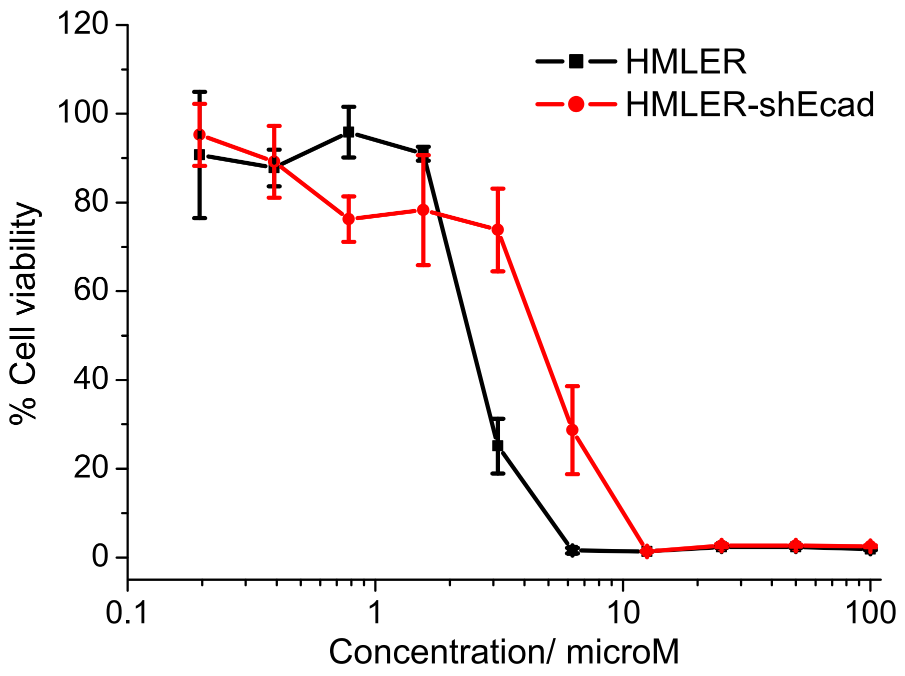

Figure S12. Representative dose-response curves for the treatment of HMLER and HMLER-shEcad cells with **1** after 72 h incubation.

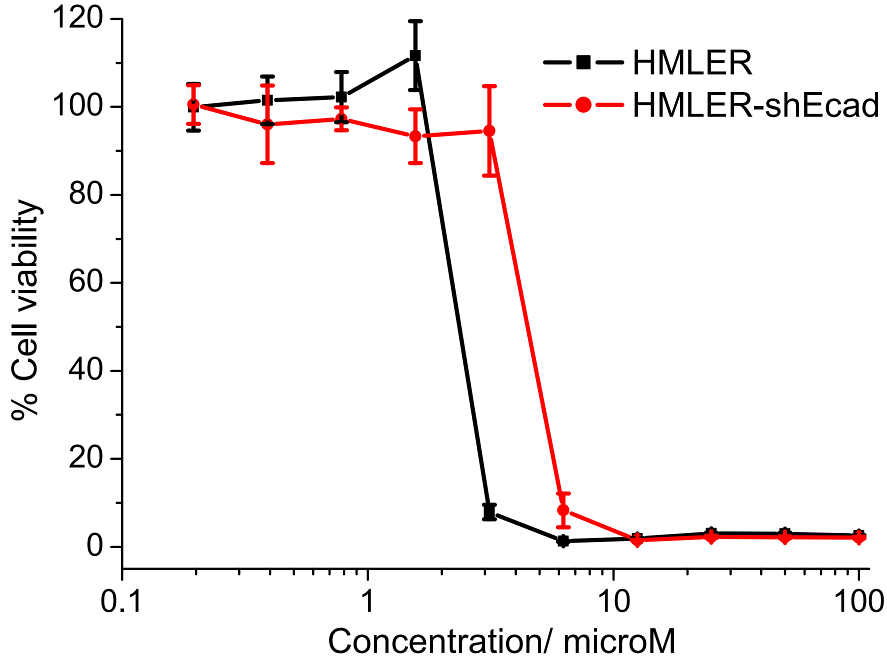

Figure S13. Representative dose-response curves for the treatment of HMLER and HMLER-shEcad cells with **2** after 72 h incubation.

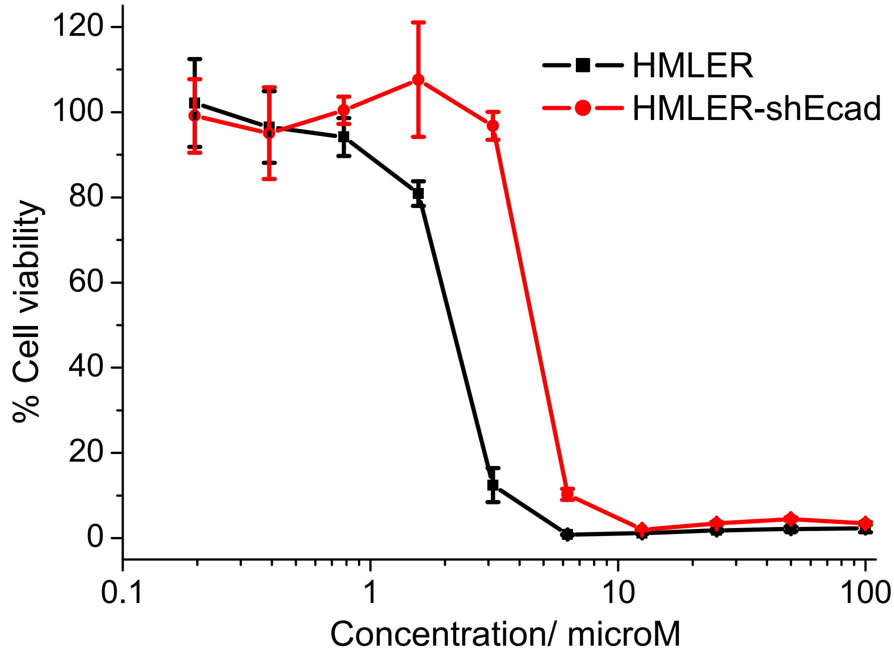

Figure S14. Representative dose-response curves for the treatment of HMLER and HMLER-shEcad cells with **3** after 72 h incubation.

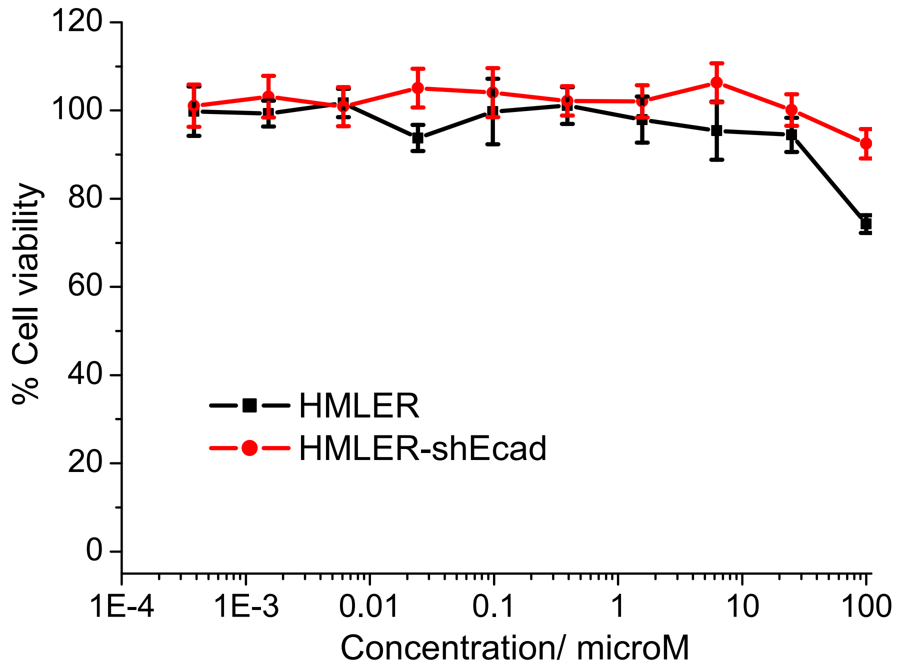

Figure S15. Representative dose-response curves for the treatment of HMLER and HMLER-shEcad cells with sodium salicylate after 72 h incubation.

Table S3. IC_{50} values of diclofenac, naproxen, and sodium salicylate against HMLER and HMLER-shEcad cells. [1]Determined after 72 h incubation (mean of three independent experiments ± SD). [2] Taken from references [1,2].

Compound	HMLER IC_{50} [μM] [1]	HMLER-shEcad IC_{50} [μM] [1]
diclofenac [2]	69.50 ± 8.70	54.74 ± 0.23
naproxen [2]	> 100	> 100
sodium salicylate	> 100	> 100

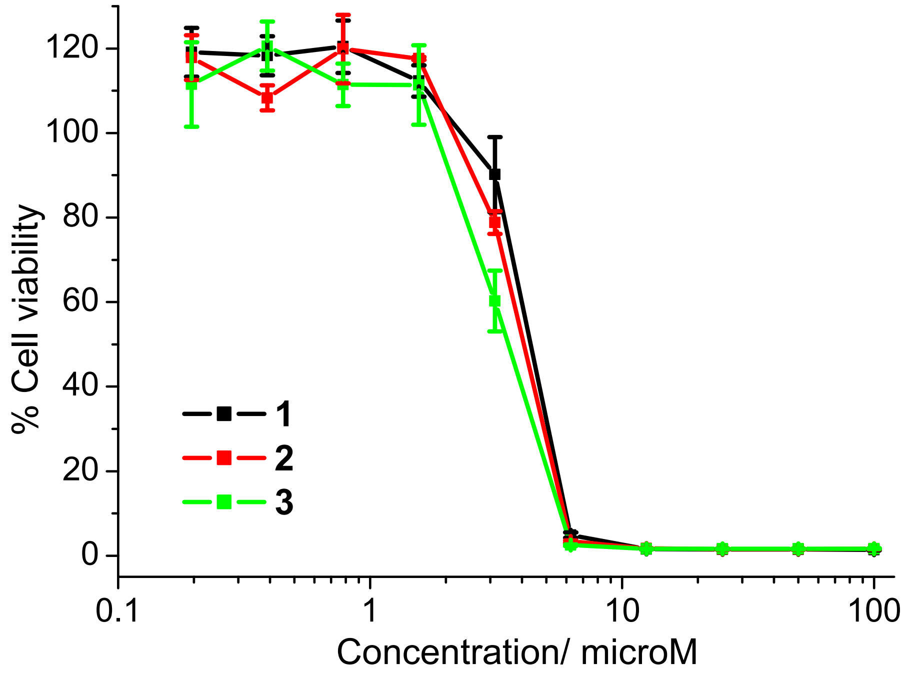

Figure S16. Representative dose-response curves for the treatment of BEAS-2B cells with **1-3** after 72 h incubation.

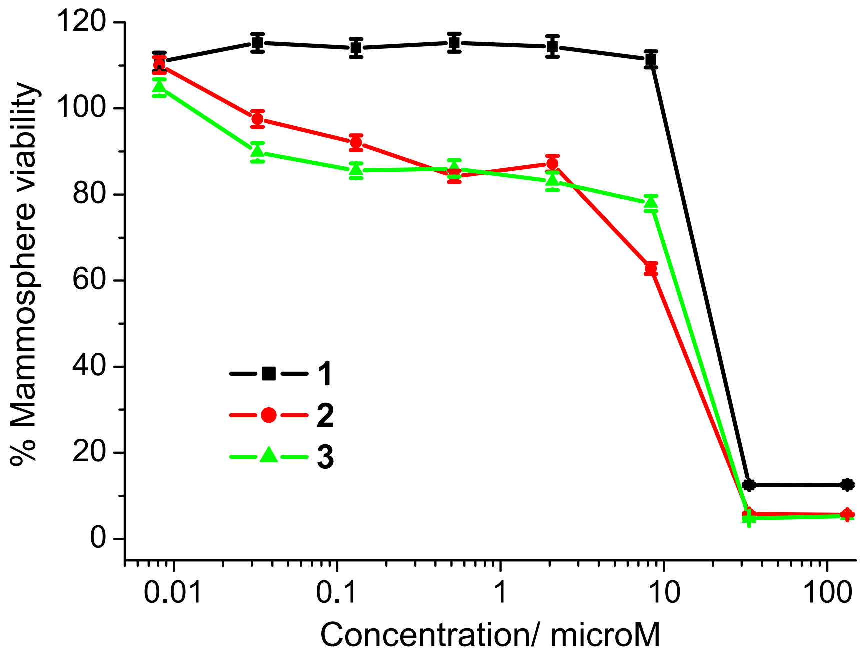

Figure S17. Representative dose-response curves for the treatment of HMLER-shEcad mammospheres with **1-3** after 5 days incubation.

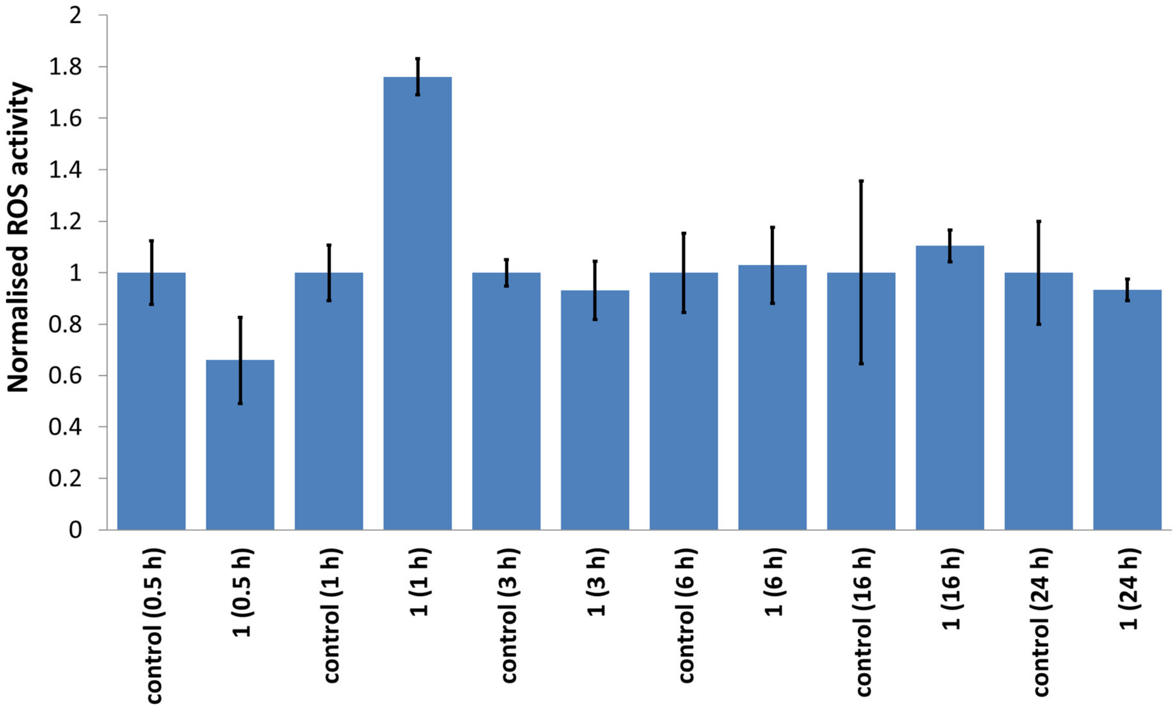

Figure S18. Normalised ROS activity in untreated HMLER-shEcad cells (control) and HMLER-shEcad cells treated with **1** (2 × IC_{50} value, 0.5-24 h).

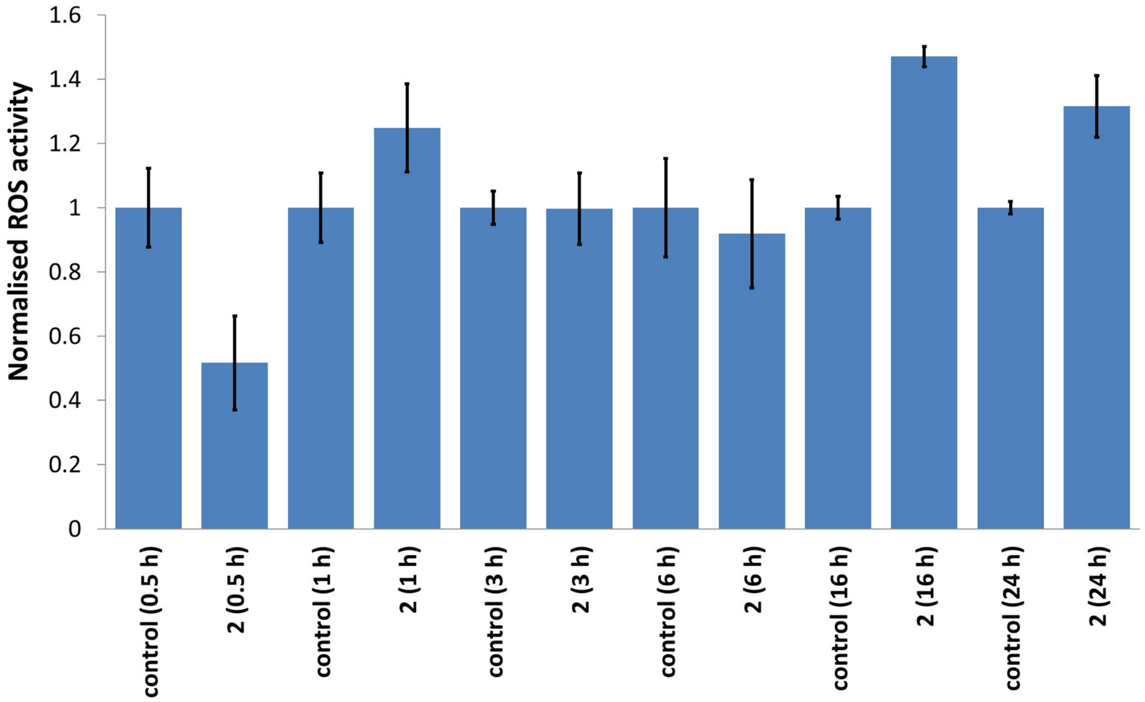

Figure S19. Normalised ROS activity in untreated HMLER-shEcad cells (control) and HMLER-shEcad cells treated with **2** (2 × IC_{50} value, 0.5-24 h).

References

1. Eskandari, A.; Boodram, J.N.; Cressey, P.B.; Lu, C.; Bruno, P.M.; Hemann, M.T.; Suntharalingam, K. The breast cancer stem cell potency of copper(II) complexes bearing nonsteroidal anti-inflammatory drugs and their encapsulation using polymeric nanoparticles. *Dalton Trans.* **2016**, *45*, 17867-17873.
2. Eskandari, A.; Suntharalingam, K. A reactive oxygen species-generating, cancer stem cell-potent manganese(II) complex and its encapsulation into polymeric nanoparticles. *Chem. Sci.* **2019**, *10*, 7792-7800.
